# Artificial Photosynthesis: Is Computation Ready for the Challenge Ahead?

**DOI:** 10.3390/nano11020299

**Published:** 2021-01-24

**Authors:** Silvio Osella

**Affiliations:** Chemical and Biological Systems Simulation Lab, Center of New Technologies, University of Warsaw, Banacha 2C, 02-097 Warsaw, Poland; s.osella@cent.uw.edu.pl

**Keywords:** multiscale computation, electron transfer, light harvesting

## Abstract

A tremendous effort is currently devoted to the generation of novel hybrid materials with enhanced electronic properties for the creation of artificial photosynthetic systems. This compelling and challenging problem is well-defined from an experimental point of view, as the design of such materials relies on combining organic materials or metals with biological systems like light harvesting and redox-active proteins. Such hybrid systems can be used, e.g., as bio-sensors, bio-fuel cells, biohybrid photoelectrochemical cells, and nanostructured photoelectronic devices. Despite these efforts, the main bottleneck is the formation of efficient interfaces between the biological and the organic/metal counterparts for efficient electron transfer (ET). It is within this aspect that computation can make the difference and improve the current understanding of the mechanisms underneath the interface formation and the charge transfer efficiency. Yet, the systems considered (i.e., light harvesting protein, self-assembly monolayer and surface assembly) are more and more complex, reaching (and often passing) the limit of current computation power. In this review, recent developments in computational methods for studying complex interfaces for artificial photosynthesis will be provided and selected cases discussed, to assess the inherent ability of computation to leave a mark in this field of research.

## 1. Introduction

The need to find new sources of energy production for our everyday consumption is one of the most pressing issues of our age. To overcome the dependence on fossil fuels and to assess renewable energy resources, innovative paths must be followed, to go beyond the conventional technologies that are already available. Moreover, considering the climate change problem and environmental pollution as a result of production and consumption of energy from fossil fuels, the need for green and renewable energy sources is a milestone which needs to be reached in the next two decades. If no necessary actions are taken, inevitably our future generations will be adversely affected. To counteract these problems, the European Union (EU) set a “Renewable Energy Directive” with a target of 55% reduction of greenhouse gas emission and reaching carbon neutrality by 2050 [1]. In the frame of these ambitious goals, the need of novel efficient materials is a priority for developing a competitive technology, with much effort already dedicated to bio-ethanol and other biofuels, novel photovoltaic technologies, including organic and perovskite solar cells, advanced batteries and supercapacitors, as well as electro- and photocatalytic systems for hydrogen production and a new class of fuel cells for transport.

A different approach to this problem is to take advantage and mimic what nature was able to perfect upon millions of years of evolution. Yet, one of the biggest challenges in modern science is to replicate in an artificial way the most basic processes performed by nature, such as photosynthesis. This fundamental process has been found to be a challenge to reproduce in laboratory settings using artificial, man-made systems/devices [2,3,4]. This is due to the many components needed from light-driven charge separation to the transport of charges between different photosynthetic reaction centers and to the final production of reducing equivalents, subsequently used for solar fuels synthesis. Although almost all of these steps are nowadays fully understood, mimicking this complex photosynthetic machinery in the full solar-to-fuel artificial devices that use water as the sole source of reducing equivalents is still prohibitive at an industrial scale. In particular, in the last five years research effort has been focused on the creation of an “artificial leaf” device that is able to produce clean energy from absorption of light, with some encouraging results [5,6]. Devices with external quantum efficiency of 30% has been reported [7], yet the overall efficiency is still below 1% [8,9] due to the cell design and lack of control over the light harvesting (LH) systems orientation.

Despite being still in its infancy, this relatively-new research approach has the potential of taking advantage of two well-known branches of research: photobiology and nanomaterial science. In fact, by combining in a synergetic way the optical and electronic properties of different nanomaterials, it might be possible to create a new class of biomimetic, hybrid devices with enhanced impact on sustainability. The potential of such collaboration is to take advantage of the best features of the two fields and design and build devices that mimic the photosynthetic apparatus of plants in a laboratory setting.

In nature, photosynthesis occurs through a highly complex apparatus in which the light is absorbed and converted into electrons via two large membrane proteins, such as photosystem I (PSI) and photosystem II (PSII) [10,11] found in higher plants as well as their counterpart (i.e., LH2, which contain a vast range of carotenoids and bacteriochlorophylls depending on the species) found in purple bacteria [12] with a multi-step proton-coupled electron transfer chain reaction that has been perfected during evolution to efficiently convert solar energy into the biological hydrogen-storage compound, reduced nicotinamide adenine dinucleotide phosphate. As a result of the sequence of reactions that take place within the photosynthetic process, the plant or alga converts solar energy into chemical energy. The key to success relies on: (1) Physical separation of the light collection (protein antenna) and the catalytic center; (2) absorption of two photons for generation of sufficient potential for water oxidation; (3) spatial separation of oxidative and reductive chemistry and relevant products; (4) multielectron character of water oxidation and proton/CO_2_ reduction; (5) efficient management of proton-coupled electron transfer for both catalytic reactions, and last but not least (6), use of self-assembled and self-renewable molecular catalysts embedded in responsive matrix [13]. Natural photosystems operate as nearly perfect photoelectrical/photovoltaic molecular nanomachines, exhibiting internal quantum efficiency close to 100% [12].

When trying to replicate this complex process in a man-made environment, where the only components are solar light, a photocatalyst, CO_2_ and water as reducing agent, the process is named artificial photosynthesis. In this way, mimicking the natural process, the light-driven conversion of water and CO_2_ leads to the formation of chemical fuels. The fabrication of such a full bioelectrode has been reported very recently [8]. It consists of highly-oriented PSI on a single layer graphene electrode involving a “sandwich” stacking structure with different biological and organic molecular linkers. To assure a high orientation of the PSI, the protein was wired to the functionalized graphene surface and genetically engineered to incorporate affinity tags (or metal-binding peptides) into their structure. Another strategy is to create intermolecular interaction between the light harvesting protein (LHP) and natural electron donors, such as cytochrome *c*553, which promotes the electron transfer to the electrode and can be used as the biological conductive interface between graphene and LHP. Yet, with this strategy an additional degree of complexity is added, since a specific orientations of the cytochrome over the electrode needs to be additionally provided. 

Despite the great experimental effort in building such complex devices, many questions are still unanswered. In particular, (i) how to effectively harvest light with small harvesting proteins and transfer the formed electrons to the redox center; (ii) how to maximize the direct electron transfer (DET) from the protein to the metal electrode, and consequently (iii) suppress charge recombination; and (iv) how to direct the charge flow to the selected targets (from material to protein or vice versa) to maximize the efficiency of the whole device. 

As the process of building the full system is lengthy due to the presence of different building blocks and the interfacing between protein and materials is not a trivial task, computation can indeed make a change in the approach for the rational design of such devices. Moreover, the availability of highly resolved structures from the field of structural biology, from X-ray crystallography, and more recently from cryo-electron microscopy (cryo-EM) allowed computation to firmly step into this field of research. As a consequence, using different computational methods in a multiscale fashion, it might be possible to reveal the mechanisms underneath these pressing questions and to predict the DET mechanism. This mission is extremely challenging, not only due to the presence of the biological light harvesting protein, but also due to the questions which the creation of such interfaces bring. In particular, three key aspects and questions can be defined: (i) How to properly describe the light harvesting process; (ii) how to maximize the charge separation at the interface, and (iii) how these separated charges can be collected at the reactive center to perform the redox reaction for the formation of solar fuels. The extreme development and power of modern computational clusters and the progress in software availability allow nowadays computation to become one of the most powerful tools to assess these challenges. 

**Figure 1:**
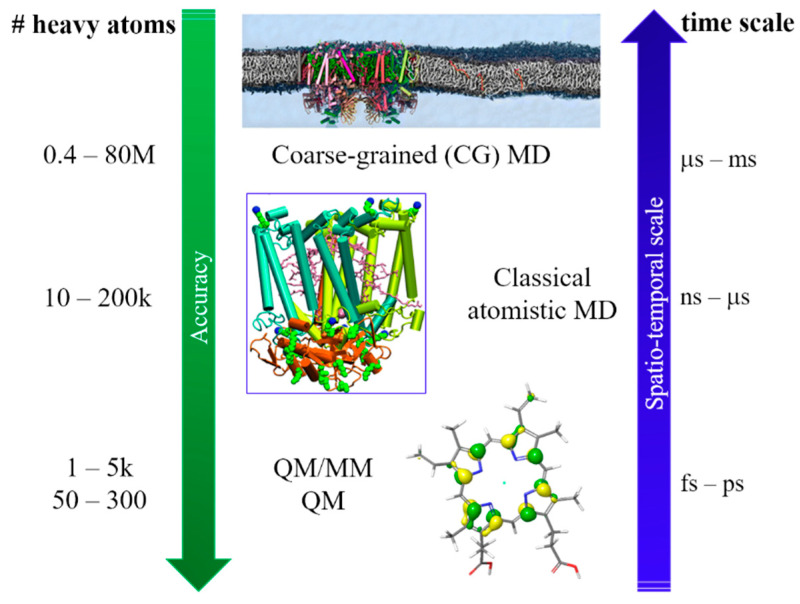
Multiscale approach for the study of photosynthetic interfaces. Different spatio-temporal resolution are reported. While the system size increases, longer simulation times are required, at the expenses of a decrease in accuracy. Adapted from [14], Springer, 2020. Abbreviations: Quantum mechanics (QM), QM/molecular mechanics (MM), and molecular dynamic (MD).

To reach these ambitious goals, computation must go beyond the traditional approach in which only a small part of the whole system was considered, and fully describe the protein and the environment (i.e., water molecules, ions). Thus, a multiscale approach is a necessity, since different properties can be described at different levels of theory. As the size of the investigated systems increase, the required simulation time increases which enforce a decrease in accuracy. In quantum mechanics (QM) and QM/molecular mechanics (MM) calculations, the nuclear and electronic degrees of freedom are explicitly considered. QM/MM methods are one of the most popular computational approach used to study biological systems and interfaces, due to the possibility of dividing the whole system into fragments described at different level of theory. Within this approach, it is possible to characterize the energy and (early) electron transfer (ET) processes considering explicitly the environment, which can have a strong impact on both energy and electrons transfer. When larger systems are considered, only nuclear degrees of freedom can be taken into account (completely neglecting the electrons) and we can now retrieve to atomistic molecular dynamic (MD) simulations. For even larger systems, MD becomes inefficient due to the short sampling time reachable and only the coarse-grained (CG) level provide reasonable simulation time. A vast variety of photosynthetic complexes and processes has been successfully investigated in recent years by applying this multiscale approach on a wide range of spatio-temporal scales, going from full atomistic to classical MD and CG methods [14] (Figure 1).

In this review, selected recent cases in which the multiscale approach is successfully applied are presented, describing the different parts and mechanisms involved in a common artificial photosynthetis system: (1) Light harvesting protein; (2) ET in the protein and (3) at the protein/material interface, showing how modern computational methods can overcome the different bottlenecks present in each aspect, and the challenges ahead. We would like to stress here that the aim of this work is not to give a comprehensive view on light harvesting only, as excellent recent reviews can be found in literature [15,16,17,18], but to give an overview on the most important processes which take place in these artificial interfaces, namely light absorption and ET. 

## 2. Light Harvesting

A vast amount of pigment–protein complex (PPC) crystal structures have been solved in the last decades, including highly symmetric systems such as light-harvesting complexes in purple bacteria or less ordered ones like the major light-harvesting complexes (LHCI and LHCII) of plants. PPCs can be considered as models for light-harvesting since both the capture of sunlight by antenna complexes (the initial step of photosynthesis) and the following transfer of the absorbed energy to the reaction centers (RCs, where charge separation is realized) are achieved with extremely high efficiencies. The mechanisms involved in the electron transfer (ET) and electronic energy transfer (EET) in natural PPCs are known [19,20,21,22,23,24,25], and there is no theoretical obstacle in building its artificial analogues. Yet, this is still difficult to obtain, particularly regarding efficiencies and stabilities required for large-scale manufacturing. It is in here that computation can be considered as optimal method to assess these challenges. In fact, real advantages can be expected if accurate molecular-based theories could be transformed into efficient computational protocols able of simulating in an accurate and comparable way natural PPCs and artificial systems. Quantum chemistry is here the method of choice due to its versatility and extensibility towards different spatio-temporal scales, especially when combined with MD simulations. The “holy grail” of such theoretical approach would be to be able to perform computation of the light-harvesting process at the QM level of theory, followed by the quantum dynamic simulations of the excited states to follow in time the photoinduced process [26,27,28,29,30]. This approach would give access to the EET dynamics and will also allow an extremely accurate simulations of the optical spectra which, in turn, dictate the spectral cross-section for light absorption, enabling a direct comparison with the experimental knowledge of natural photosynthetic complexes. Unfortunately, the complexity of LH systems and the size of the network of interacting chromophores have so-far precluded the direct application of this approach. Despite this drawback, computation can still be efficiently used with QM/MM. This method combines the description of the chromophore at the QM level and the classical description of the environment. The success of this method rely in the explicit description of the interactions between the two parts. The classical part is generally divided into two families, depending on the type of description considered for the environment, namely as a continuum or with explicit account of the atomic degree of freedoms by using MM forcefields. The QM part can be also be described in its electronic and nuclear terms due to the presence of the classical part. The total Hamiltonian becomes:

(1) $$H=H_{\mathrm{QM}}+H_{\mathrm{env}}+H_{\mathrm{int}}$$

where $H_{\mathrm{QM}}$ is the Hamiltonian of the isolated chromophore (in vacuum), $H_{\mathrm{env}}$ of the rest of the system expressed at the classical level and $H_{\mathrm{int}}$ of the interactions between the two. In both QM and classical descriptions the degrees of freedom of the environment are only marginally important, while in the continuum solvation model (in which the atomistic description disappears) this term disappears. $H_{\mathrm{env}}$ is maintained for QM/MM approach as a constant term to the total energy. The most common formulation of the QM/MM approach considered the MM part described as a set of fixed point charges placed at the atoms positions of the environments. It is thus evident that to accurately describe light-harvesting processes in both artificial and natural systems, simplification should be taken with care, as completely neglecting the environment will lead to inaccurate descriptions of the processes of light harvesting and EET. Different strategies have been developed over the years to integrate the environment within a quantum chemical description of an embedded molecule. The most computationally efficient and straightforward approach is to consider the environment as an infinite continuum dielectric (i.e., polarizable continuum model, PCM). By adding a surface charge distribution to model an induced polarization, mutual influence of the environment over the QM part is obtained [31]. The definition of the cavity in which the QM part is immersed becomes the key parameter for this method. This approximation results in very good ratio between accuracy and computational cost, explaining its popularity. In cases when specific interactions between the QM part and selected atoms of the environment are important (such as van der Waals, electrostatic, and hydrogen bonding) this approach fails, and an atomistic description of the environment must be introduced. In such cases the method of choice is QM/MM, in which the environment is described through molecular mechanics (MM) force fields [32]. This lead to electrostatic interactions only between the QM and MM parts, in which the MM charges represent the molecular electrostatic properties of the environment. Yet, more sophisticated methods can be considered to describe the MM part, namely with higher multipole moments, such as dipoles, in which the polarization of the environment is additionally taken into account. 

The widely used electrostatic embedding scheme considers the inclusion of the effect of the MM atoms in the QM Hamiltonian, having the same form of the Coulomb term [33]. The interaction term of the Hamiltonian can be thus defined as:

(2) $$H_{\mathrm{int}}=\underset{i}{\sum }q_{i}V_{\mathrm{QM}}\left(r_{i}\right)$$

where $q_{i}$ are the partial charges at position $r_{i}$ and $V_{\mathrm{QM}}\left(r_{i}\right)$ is the electrostatic potential operator due to the QM charge density. In this scheme, the electronic structure of the QM part is modified by the presence of the MM charges representing the environment and its polarized by it. Yet, the opposite scenario (MM term polarized by the QM one) is not present and this is the main limitation of the electrostatic embedding, as the explicit response of the environment might be of extreme importance when polar environment are present or when excitation processes are considered. Despite this main drawback, the electrostatic embedding is still widely applied, especially because of the short computational time required, when compared to more sophisticated descriptions. Nevertheless, recently different improvements started to be used also for the study of LH systems. A step forward in a more accurate description of the environment effect on the QM properties is the polarizable embedding, in which mutual polarization between the classical MM and the QM parts is explicitly included. In particular, we refer here to the induced dipole model in which polarizabilities are introduced. In this way, dipoles are present on both QM and MM parts and interact with each other, leading to a self-consistent procedure to solve this QM/MM interactions. As consequence, an additional parameter is added to the Hamiltonian in Equation (2), namely the atomic polarizabilities, defined as a 3 × 3 tensor [16]. The main drawback of this last method is the high computational cost to pay for accounting of these interactions, for which the computation of the polarizabilities term is the most computationally time consuming. 

A recent development consists on the merging of MM and continuum approaches to give fully polarizable QM/MM, in which in addition to the polarizable model described above an external polarizable continuum layer coupled to the former is added. In such a way, a three layers model is obtained in which the polarizable QM/MM (used to describe directional, short-range interactions) is combined with the additional continuum description of the environment (used to describe long-range, bulk effects), taking the advantages of both methods [34]. 

As the response of the environment to dynamic is slower compared to the electronic motion, fast and slow components should be considered during absorption or emission processes, respectively; only the fast component can completely relax after changes in the QM electronic density, while the slow component is kept frozen. This partition describes the nonequilibrium regime, and in particular for highly polar environments the different configurations arising from equilibrium and non-equilibrium regimes cannot be neglected, and this energy difference is known as reorganization energy. In QM/continuum approach, this is reached by separating the optical (fast response) and static components (slow response) of the dielectric constant, while in the QM/MM approach, in which the environment is explicitly accounted for, the nuclei represent the slow component while the polarizability accounts for the fast component. Note here that in the electrostatic embedding scheme only the slow response can be considered, as the MM charges are fixed at the position of the nuclei.

For what concerns the chromophore (the $H_{\mathrm{QM}}$ part in Equation (1)), the description is more complex. In fact, they are commonly present as aggregates in LHCs, and the electronic coupling between them shall be considered too. For this reason, the description of the excitons are based on Frenkel model [35], for which the single exciton can be expressed by the following Hamiltonian:

(3) $$H_{e}=\;\underset{i}{\sum }E_{i}|s_{i}\rangle \langle s_{i}|+\underset{i}{\sum }\underset{i\neq j}{\sum }V_{ij}|s_{i}\rangle \langle s_{j}|$$

where $|s_{i}\rangle$ represents the exciton localized on a site i (i.e., a single chromophore in a LH system) with energy *E*_i_. $V_{ij}$ is the electronic coupling between local excitons states $|s_{i}\rangle$ and $\langle s_{j}|$. The Hamiltonian of the total aggregate is thus the sum of single Hamiltonians. Generally the second term in Equation (3) is a sum of Coulomb and exchange interactions between the two sites, and is one of the key quantity to be computed to determine the EET rates and mechanism. Moreover, in the Frenkel exciton description, the $|s_{i}\rangle$ states form orthogonal basis. This assumption holds true for LH systems, in which the chromophores (i.e., different pigments) are locate at a distance such as to have negligible overlap integrals [16]. Yet, in this model the delocalization is considered only as superposition of local exciton states, determined by the magnitude of $V_{ij}$ and by the *E*_i_-*E*_j_ energy difference, completely neglecting important terms such as polarization and vibration response arising from the protein environment, which should be included in the Hamiltonian. The most used approximation for the Coulomb term in the electronic coupling to the environment is the point transition dipole approximation [16] in which this term is considered as interaction of transition dipole moments. This approximation performs well at large distances (as found in between chromophores of LH systems) while it breaks down at close distances, where the molecular geometry description becomes important. A more robust approach which overcomes these limitation considers the computation of transition densities [36] which can be obtained from different methods, from TDDFT to configuration interaction with single excitations (CIS), second-order approximate coupled cluster (CC2), complete-active-space self-consistent-field (CASSCF), and equation of motion coupled cluster (EOM-CC) [37,38,39,40]. These last methods are much more computational expensive for what concerns time and computational resources, compared to the point dipole approximation, and are still often neglected, despite the great importance they hold.

To take into account the effect of the surrounding of the chromophore, and overcome the aforementioned limitations, additional terms in the electronic Hamiltonian must be added, to account for factors such as vibrations and polarization response of the protein environment. The total Hamiltonian for the QM part in Equations (1) and (3) thus must be rewritten to include the exciton ($H_{e})$, the environment (or bath, $H_{b}$) and the interaction between the two parts ($H_{\mathrm{be}}$):

(4) $$H_{\mathrm{QM}}=\;H_{e}+H_{b}+H_{\mathrm{be}}.$$

In this expression, all the degree of freedoms coupled to the exciton states, such as nuclear degrees of freedom of the protein residues and pigment molecules, are considered, as well as the electronic degrees of freedom not directly involved in the electronic excitations of chromophores. All these terms are needed for a quantitative characterization of the excitons, to obtain a reliable assessment of the migration mechanism of the excitons from one chromophore to another in the LHCs, and paving the way to a general design principle for each LHC. The bath-electron Hamiltonian ($H_{\mathrm{be}}$) is often considered as a set of harmonic oscillators, in which only diagonal terms are considered, while off-diagonal terms are assumed to be negligible. For a detailed review on the theoretical derivation of these terms, together with a detailed description of the spectroscopic observables which can be derived, we refer here to [16].

Depending on the strength of both the electronic coupling and the coupling to the environment, the EET rate can be described either by the Förster regime in which the square of the electronic coupling is assumed to be much weaker than the reorganization energy, or by the Redfield regime where the excitonic coupling are much larger than the coupling to the environment. In the last case the process is dominated by relaxation and not EET. We would like to stress here that these regimes are extreme cases, and natural and artificial PPCs are often found in between them, reaching maximum efficiency [41,42]. A detailed description of both regimes lies outside the scope of this review, but we indicate here a few literature references in which both methods are discussed in details [12,16,43,44]. What we would like to stress in here is the different type of systems for which either one or the other description is applicable. In fact, while the Förster theory can be applied for chromophores which are distant from each other (more than 1.4 nm) for which the coupling is weak, leading to a localization of the excitons, the Redfield theory should be used when at least a partial delocalization of photosynthetic excitons is present, and chromophores are strongly coupled, for which the transfer between weakly coupled aggregates with strong intra-aggregate excitonic couplings is present. However, many complexes are in the intermediate coupling regime that can be described by numerically exact approaches for which appropriate methods as generalized Förster theory exists [12]. In this intermediate regime, the accurate computation of quantum dissipative dynamics are obtained with nonperturbative numerical methods [45,46]. These methods are of particular interest when the excitation localized when the nuclei relax (described as dynamic localization). The hierarchically coupled equations of motion (HEOM) [45] and other nonperturbative approaches on the other hand treat in principle all couplings exactly. However, many quantum chemical studies on multichromophoric complexes only deal with the electronic part, computing site energies and exciton couplings, due to the complexity of the suggested methods and the high computational time required.

In this section, different approaches describing the EET processes are presented, focusing on QM/MM, network modeling and post HF methods nowadays available. The section ends with a short view on the TTET and the challenges present when dealing with spin forbidden transitions. For an exhaustive review on the use of different computational methods to simulate light harvesting systems, we refer to [15,16,17,18].

### 2.1. QM/MM-Based Approaches

**Figure 2:**
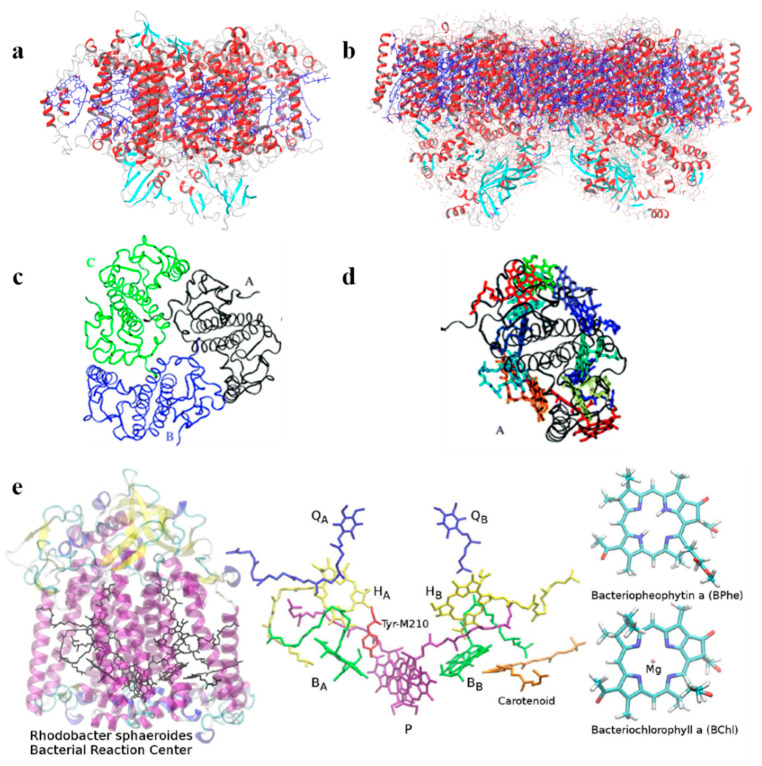
Structure of photosystem I (PSI, (**a**)) and photosystem II (PSII, (**b**)) from pdb entry 1JB0 and 3WU2, respectively. Trimeric light-harvesting complex (LHC II) complex from pdb entry 2BHW; the different chains are indicated by different colors (**c**); chlorophyll residues in chain A, in which the Chl residues are colored in yellow and red tones (**d**). Reproduced from [47] with permission from The Royal Society of Chemistry, 2011. Representation of the BRC of Rhodobacter sphaeroides based on X-ray study (**e**). The core pigment units are the P, and the pairs of BChl, and BPhe, shown in the central part along with representation of their neighbouring pigments that form the distinct effective dielectric for the two branches. Reprinted with permission from [50]. Copyright (2019) American Chemical Society.

As early as 2011, König et al. [47] considered a QM/MM method using various (TD)-DFT approaches to describe the absorption properties of LHCII and chlorophyll pigments (Chl) of green plants while explicitly consider environmental effects (Figure 2c,d). The protein environment is here considered as “test environments” in which only the amino acids closest to the QM region are considered. The response of the environment to the changes in charge distribution of the pigments during excitation have been accounted for by use of polarizable models, in which the environment is considered classically, as described in the generalization of the frozen-density-embedding (FDE) for excited states [48,49] in which the environment is considered at the QM level, without system-specific parametrization. With this methodology, the authors calculated the changes of site energies of all chlorophyll pigments in LHC II and the absorption spectrum of the complete chlorophyll network (the total system has more than 1000 atoms). They concluded that while carrying partial optimization with some structural constrains, the protein environment should be explicitly taken into account. Alternatively, sampling can be done over a number of different conformations of the pigments in the protein. From these calculations, the authors compute the absorption spectra of the complete Chl network and identified which pigment mediates the electronic coupling mixing (acting as bridges) between other pigments. Thus, this TD-DFT FDE based approach allows to investigate environmental and exciton-coupling effects of a network of pigments in protein complexes in a full QM treatment beyond the standard Kohn–Sham approach.

Recently, Aksu et al. [50] considered a newly developed polarization-consistent framework [51] which combines PCM with TD-DFT by using a screened range-separated hybrid functional (SRSH) to assess the absorption properties of the bacterial reaction center (BRC, Figure 2e). This method is computationally efficient and accurate for obtaining both ground and excited state properties of molecular systems in which polarization effects should be considered. The authors analyzed the spectral splitting of pairs of pigments and found that the spectral shift is due to both structural differences and the dielectric environment surrounding the pigments. They conclude that the electronic states of the core pigments of BRC are localized over individual units, and the coupling between pigments is negligible. In addition, they addressed the spectral asymmetry of BRC associated with pseudo-symmetric pairs. SRSH-PCM calculations have been performed combining different dielectric constants assigned to the specific pigment pairs, resulting in a very accurate description of the shifts in excitation energies, which are not found when neglecting the environment.

Transition dipole moments are commonly obtained from TD-DFT methods by solving the linear response Casida equation [52], for which the ground state Kohn−Sham (KS) wave function has to be computed. Despite its wide use, this procedure has two main drawbacks: (i) The solution of the Casida equation become unfeasible for large molecules/complex systems for which a large set of KS orbitals are required, and (ii) the lack of convergence for high lying virtual KS orbitals lead to the failure in describing high energy excitations. Among the variety of developed approaches, the real-time propagation TD-DFT (P-TDDFT) provides an interesting alternative to TD-DFT [53]. It has been verified that P-TDDFT is an excellent platform for studies, among others, of molecular and electron dynamics [54], (non)-linear optics [55] and transport properties [56]. In addition, P-TDDFT allows for the computation of all excitation frequencies at the cost of converging only the occupied ground state KS states [57,58], strongly reducing the computational cost and allowing the study of systems up to several thousand atoms [59,60]. These features open the way to the study of large biological systems such as LHC, while explicitly accounting for the environment and treating it at the same atomistic level of theory [61]. Within this approach, the transition dipole moments and transition densities of the individual pigments can be obtained, permitting the computation of a broad excited states’ energy range without neglecting the effects induced by the environment.

**Figure 3:**
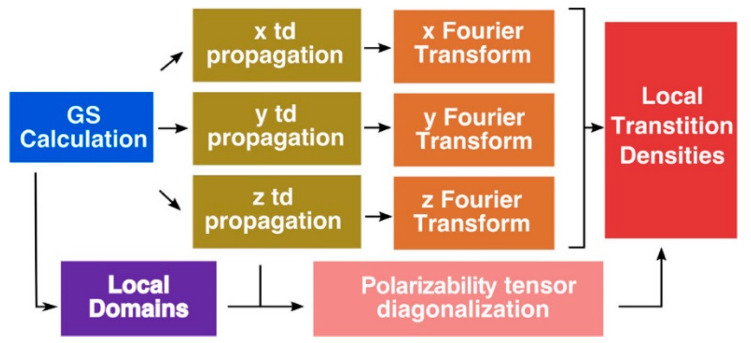
Scheme of the procedure used to get transition dipole moments and transition densities from real-time propagation TD-DFT (P-TDDFT) calculations for multichromophore systems. Reprinted with permission from [53]. Copyright (2019) American Chemical Society (https://pubs.acs.org/doi/abs/10.1021/acs.jctc.9b00209. Further permissions related to the material excerpted should be directed to the ACS).

Jornet-Somoza et al. [53] applied P-TDDFT to LHC systems, considering all the photoactive chromophores at the same ab-initio level of theory, following the procedure reported in Figure 3. Despite the inherent problem of TDDFT in properly describe CT states, the authors obtained accurate results especially for weakly interacting (large) systems by explicitly accounting for quantum mechanical environment effects. Moreover, they introduced a local density analysis to determine the transition properties of multichromophore systems. In detail, this procedure permit to extract accurate transition dipole moments and densities for an individual chromophore, while taking into account the different effects of the environment surrounding it, without any approximation (the whole system is considered at the same ab-initio level of theory). Thanks to this approach the excitonic coupling, a key parameter for EET process, can be accurately computed, as well as the exciton transfer.

Recently, due to the enhancement in computational power, the QM/MM approach has been strongly revaluated to not only consider polarizable embedding in the MM part, but also to improve the QM description by going beyond the mono-configurational picture which is behind DFT methods. Segatta et al. developed a hybrid QM/MM model which accounts for the effect of the environment when studying LH2 [62]. In particular, they consider two levels of QM theory: TD-DFT and RASSCF/RASPT2 methodology [63,64] with different Active Spaces and State Average settings in the different spectral regions. When TD-DFT is considered, both electrostatic and polarizable embedding have been considered for the MM part, while for the more computational expensive RAS calculations the charges were considered within the electrostatic embedding. For the LH2 system, in which pigments in the Qy region are strongly coupled, the authors considered the Redfield regime for the disordered exciton model [43,65], while for the Car-Ox region the Förster theory has been considered, as the reorganization energy is large in this region and the energy gap between Car and Ox is considerable. In addition, the 2D excited state pump-probe spectra were simulated with the Sum Over States (SOS) approach [66,67] using the transition dipole moments, couplings and site energy as obtained from the QM part (either TD-DFT or RAS). 

**Figure 4:**
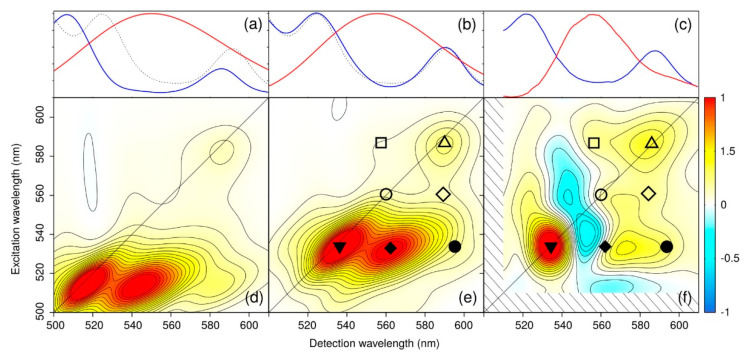
Comparison of simulated (**d**,**e**) and experimental (**f**) 2DES pump-probe maps of Rps. acidophila at 300 K and waiting time *t*_2_ = 22 fs. The corresponding linear absorptions (blue) and pulse shapes (red), along with the relative experimental linear spectra (dashed line), are reported in (**a**–**c**), respectively. In the calculated maps the pulse shape was adapted to have the same overlap between pulses and absorption bands as the one reported in the experiments. Panel (**e**) refers to a calculated map where Car *S*_2_ site energies and *Q*_x_ transition dipoles have been scaled to match the position and the intensities measured in the linear spectrum. Simulated maps are normalized to their maximum. By convention, bleach and stimulated emission contributions appear as positive (red) signals, excited state absorption appears as negative (blue) peaks. Full (referring to Cars) and empty (referring to BChls) geometric symbols are used to indicate correlations between calculated and measured signals. Reprinted with permission from [62]. Copyright (2017) American Chemical Society.

With this novel approach, the authors were able to simulate both linear and 2D spectra over the Qy and Car-Ox regions, without relying on simplified descriptions of the environment or approximated QM methods. Both single and multireference QM methods have been used together with electrostatic and polarizable MM embeddings in order to properly describe the whole protein. As results, the authors showed that, in order to reproduce accurate 2D maps, the pulse envelops must be included in the simulations. As result of this complex and highly accurate methodology, they were able to simulate several observed signals in the 2D ES maps, and indicate that there is a contribution form a dark state which was predicted by experimental observations but not yet observed by computations (Figure 4). Moreover, this approach shows that the integration of multiscale models and experiments is crucial to reveal the complex network of ET routes in LH2.

**Figure 5:**
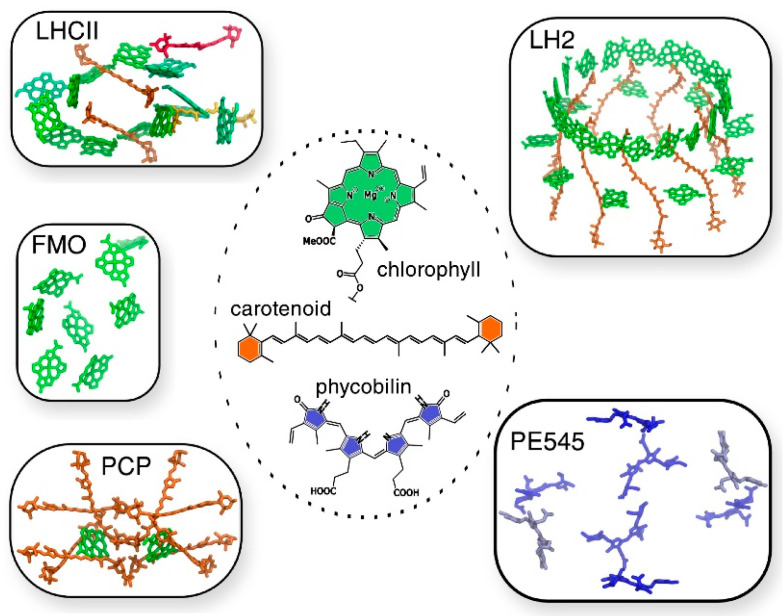
Examples of multichromophoric aggregates as found in different LH complexes. Starting from the left upper corner we see the major LH complex of plants LHCII (here represented in its monomeric form), the LH2 from *Rhodoblastus acidophilus* (purple non-sulfur bacteria), the phycoerythrin 545 (PE545) antenna of *Rhodomonas* sp. (marine algae), the peridinin–chlorophyll a–protein (PCP) from *Amphidinium carterae* (oceanic plankton) and the Fenna–Matthews–Olson (FMO) complex of *Prosthecochloris aestuarii* (green sulfur bacteria). In the central ellipse a schematic representation of the different types of pigments is reported; namely (bacterio)chlorophylls, carotenoids and bilins. Reprinted with permission from [68]. Copyright (2020) Elsevier.

The same authors reported state-of-the-art methodologies in a subsequent study, considering multichromophoric aggregates of different LH complexes of different origins, in which different families of pigments, such as (bacterio)chlorophylls, carotenoids, and phycobilins, are involved (Figure 5) [68]. In this study the authors presented a common strategy to assess not only the different chemical nature of all the pigments, but also their spatial arrangement. In this computational approach, the strategy should be general enough to be applied to the variety of different pigment families, but at the same time should be tunable, to adapt to the peculiarity of each family. The real challenge faced in this work is the atomistic modeling of the whole photosynthetic light-harvesting complex, due to the very large dimensions, the strong interaction in between the pigments and the presence of the coupling within the different parts, which cannot be neglected. As the amount of pigment is significant and the degree of conjugation is high, the choice of QM methods to describe the chromophores is rather limited to TD-DFT methods. Yet, its bottlenecks (inaccurate description of CT states and impossibility to describe multiconfigurational character excitations) should always be remembered. 

**Figure 6:**
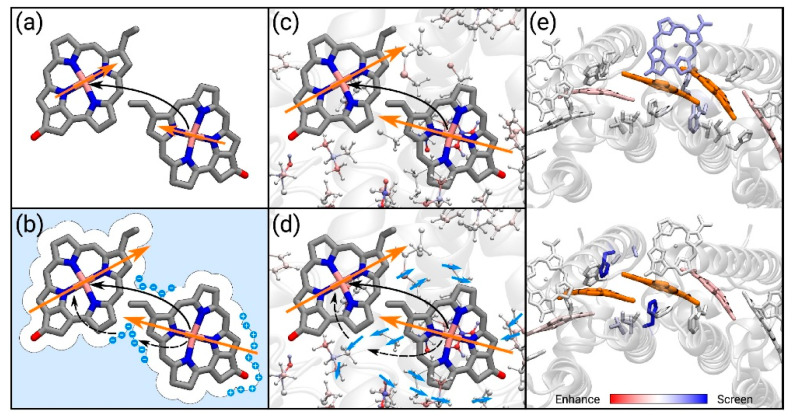
(**a**) Electronic coupling computed in vacuo. The interaction is between the two transition dipoles. (**b**) Electronic coupling computed in a polarizable continuum scheme. The transition dipoles are enhanced by the environment and, in addition to the dipole–dipole interaction (solid arrow), there is an interaction through the environment (dashed arrows). (**c**) Electronic coupling computed in a MM electrostatic embedding. The transition dipoles are enhanced by the environment, but there is no screening effect. (**d**) Electronic coupling computed in a MM polarizable embedding (MMPol). The transition dipoles are enhanced and there is an interaction through the environment. (**e**) Effect of the polarizable environment on two non-equivalent electronic coupling of the LH2 (blue: Screening, red: Enhancing). Note how the histidines coordinating the BChls reduce the second coupling while they do not affect the first. Reprinted with permission from [68]. Copyright (2020) Elsevier.

As previously described, when considering atomistic models such as QM/MM, the environment is often considered either with the electrostatic or the polarizable embedding [69]. A different, more rigorous approach considers the use of the frozen density embedding scheme (FDE) [70], in which the system is divided into a number of fragments each described at the QM level, but only one of them is kept free to move, while all the other are frozen [71], thus completely eliminating the classical description of the environment. Although the computational cost of this approach is higher, the advantage is the ability of explicitly obtaining the electronic exchange and overlap terms, and not to add them to the Coulomb term of the Hamiltonian as commonly done [72]. With the presence of the protein affecting the coupling, different mechanisms should be considered [73], either through the effect of the transition densities of the dimers (implicit mechanism) or by screening of the Coulomb interaction (explicit mechanism), Figure 6.

Considering all these interactions explicitly, without introducing any approximation related to the methods used, the authors were able to reveal the delicate interplay of the different mechanisms over all the components on LH systems. In this impressive state-of-the-art work, the authors reported novel strategies suitable for simulating the dynamic of real systems with tens of thousands of atoms at a very high level of accuracy, taking into account all the different scales of the components in a very coherent way. This study really pushed the boundary of computation beyond the state-of-the-art, opening novel, exciting perspectives on the integration of new theoretical models and algorithms, as well as new tools for the analyses.

Kim et al. [74] applied a different QM/MM approach to study the Fenna−Matthews−Olson (FMO) pigment−protein complex. FMO is one of the most studied LH complex, present in green sulfur bacteria which primary function is to transfer the excitation energy form the antenna complex to the reaction center, where the charge transfer process is initiated. FMO is a trimer in which each subunit possess eight bacteriochlorophyll a (BChl a) chromophores. Its structural simplicity and the complex excitonic structure makes FMO an ideal system for testing novel computational techniques.

**Figure 7:**
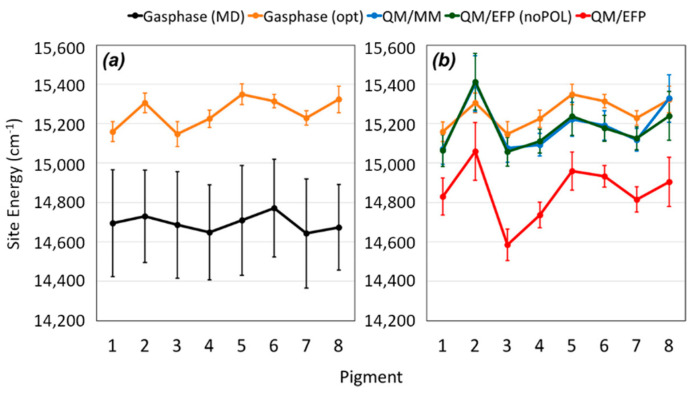
BChl a site energies averaged over 100 structures with standard deviations shown as vertical error bars. (**a**) Gas-phase (without protein environment) site energies computed for structures directly extracted from MD snapshots (black) and after QM/MM geometry optimizations (orange). (**b**) Gas phase (orange), QM/MM (blue), QM/EFP-noPOL (green), and QM/EFP (red) site energies computed for structures after QM/MM geometry optimizations. Reprinted with permission from [74]. Copyright (2020) American Chemical Society.

The authors of this study reported a comprehensive multiscale approach to reproduce the absorption and circular dichroism spectra without any input from experiment, apart the crystal structure of the complex itself, in a “single-blind” like approach. They showed that using the QM/MM together with the effective fragment potential (EFP) model to treat the environment while including polarization is the key to properly assess the excitonic structure. To reach this conclusion, the authors developed a computational protocol in which the FMO complex is fist immersed in water and equilibrated via several MD simulations, then constrained geometry optimization at the QM/MM level are computed to remove inaccuracies caused by inaccurate force field description. Next, excited state calculations with the polarizable embedding QM/EFP scheme [75,76,77] were performed, to obtain site energies and transition charges (used to compute the electronic couplings) of the pigments. We would like to remind here that the described methodology is still debated, especially the constrained optimization, as it has been reported that it removes the benefit of performing MD simulations, and has been suggested that a thorough force field parameterization to avoid this step should instead be considered. However, due to the lengthily and costly process of reparameterization, the strategy implemented by the authors can still be considered beneficial to lead to accurate results (Figure 7). 

As the authors of this study also remarked, the QM/MM is one of the most critical step in the whole procedure, together with the excited state calculations in the polarizable embedding, due to (i) limitations of the classical force field and (ii) large pigment fluctuations. Moreover, they provide evidence of the effect of polarizable MM over the site energies values (Figure 7), demonstrating the importance of correcting the pigment structures are the QM level before excited state analysis. In addition, the QM/EFP method provides a rigorous description of intermolecular interactions, without further parameterizations needed [78,79,80], in which the system is divided in rigid fragments and the interaction between them is the sum of different terms such as polarization, dispersion, Coulomb and exchange-repulsion [80,81,82], which allow for the study of highly anisotropic systems such as LHP. We remark here that the QM/EFP model used is not the same as QM/MMPol, because in QM/EFP the polarization is treated as an effective 1-electron operator, and it uses both polarizabilities and multipoles to account for self-consistent polarization. Within this approach, the excitonic properties of the FMO complex resulted in an excellent quantitative agreement with experiments, and also previous empirical computations. This is particularly impressive for the circular dichroism spectra, for which all the major features were reproduced.

### 2.2. Numerical Modeling

A different approach (not considering QM/MM method as discussed in the previous section) to study the EET in small PPCs has been recently proposed by Baker and Habershon [83]. They opted for a view in which the PPC is considered as a series of interconnected nodes (i.e., the pigments) and edges (i.e., the electronic couplings between the pigments), forming together the PPC electronic subsystem. In this approach, the Hamiltonian is equivalent to the typical tight-binding model of n interacting molecules [41,84,85]. The proposed protocol comprises Hamiltonian and dynamics, in which the Haken–Strobl model was used to consider the environment as a pure dephasing effect in the pigment excitation energies. The Hamiltonian reported in Equation (1) is modified to include the exciton recombination effects ${\hat{H}}_{\mathrm{rec}}$ and energy trapping (electrons reach the “sink” pigment and then flows out towards the RC) ${\hat{H}}_{\mathrm{trap}}$:

(5) $${\hat{H}}_{rec}=-i\hbar \Gamma \sum_{i=1}^{n}\left|i\rangle \langle i\right|\;\;;\;\;\;{\hat{H}}_{trap}=-i\hbar \kappa \left|k\rangle \langle k\right|$$

The recombination operator acts on all pigments while the trapping operator only acts at pigment *k* (PPC “sink” site). With this method it is possible to simulate the EET dynamics including both the electronic and the environmental parts, within the master equation approach described by the Haken–Strobl model [41,84,85].

It is important to note that explicit correlations between the electronic Hamiltonian and the environment are missing and that the protein is considered separately as Markovian fluctuations in the pigments’ excitation energies, leading to dephasing effect at each pigment node. Despite the different approach followed, the presented model reproduces in a quantitative way experimental results for complex systems such as excitonic energies of FMO complex and LHC-II. As in the other methods described earlier in this review, the authors found that the key parameter for the efficiency of the EET is the protein–pigment interaction. The authors considered in their study FMO and LHC-II as model cases, and demonstrated that the description of these complexes as simple network of nodes and edges qualitatively reproduces the dynamical behavior of the whole proteins. Moreover, it is possible to identify different paths of the PPCs structure to the protein light-harvesting abilities and unravel the fundamental nodes needed to obtain an efficient ET, by systematic removing pigment. It is easy to foresee that this kind of analysis can be employed for the “screening” step in the design of novel networks of chromophores like the one used in artificial solar cells or photoinduced catalytic systems. Despite the qualitative nature of this proposed approach, the computation is robust and allows the performance of fast quantum dynamic calculations on large systems. The next challenge would be to explicitly account for the pigment-environment coupling, in a computationally-simple manner.

**Figure 8:**
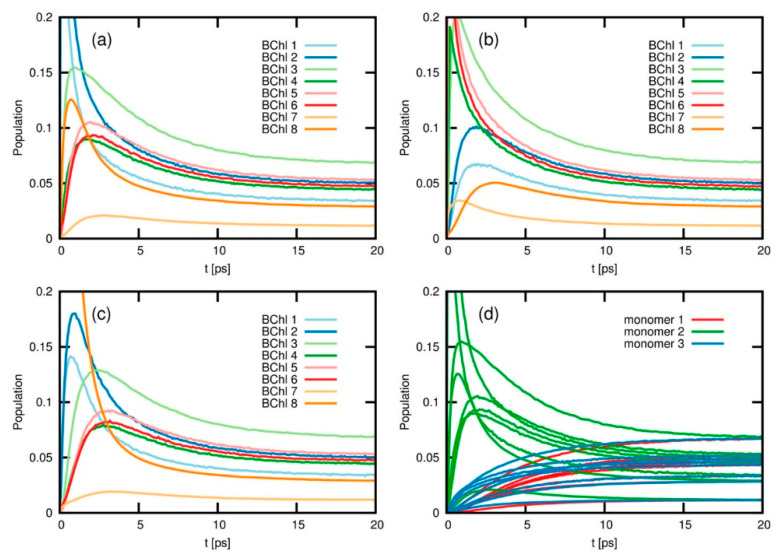
Long time electronic energy transfer (EET) dynamics in the 24-state model of the FMO trimer complex (*T* = 300 K) for initial excitation of BChl 1 (**a**), BChl 6 (**b**), and BChl 8 (**c**), respectively. The population of all 24 states is shown for initial excitation of BChl 1 of FMO monomer 2 (**d**); *k*_eff_ = 8, = 10^−7^. Reproduced from [86] with permission from AIP, 2017.

A different methodology for the simulation of energy- and charge-transfer processes is based on the iterative quasi-adiabatic propagator path integral (iQUAPI) approach [86]. This method relies on an improved numerical exact path integral quantum dynamics merging performance via mask-assisted coarse graining of Feynman-Vernon influence coefficients (MACGIC-iQUAPI). Thus, it allows for simulation of charge and energy transfer dynamics of extended systems in the intermediate regime of system bath coupling, in which Born–Markov approximation cannot be used, with convergence towards numerically exact results. It has been tested on benchmark problems ranging from simple 2-state spin-boson models up to fully coupled 24-state models of the FMO complex. MACGIC-iQUAPI is able to successfully cover parameter regimes of coherent and incoherent excitation energy transfer in dissipative quantum systems subject to classical environment with long-time bath memory as well as charge-transfer dynamics including quantum phenomena such as super-exchange on the same footing [87]. Its application to FMO complexes is of particular interest in the present context, as it was possible to obtain the long time EET equilibration dynamics on the FMO trimer and study the population of different excited states after 20 ps (Figure 8). As result, the authors observed that depending on the initial excitation conditions, different times are needed for the population to equilibrate, and this is strongly dependent on the nature of the coupling between the chromophores, going from weak (Redfield limit) to intermediate and strong (Förster limit) system bath regimes. The authors also indicate that the MACGIC-iQUAPI algorithm is useful in the description of EET coherent dynamics coupled to steady state equilibration in the Förster ET regime. 

### 2.3. Post HF Methods 

As shown in the previous sections, the majority of computational methods used to describe the PPCs electron transfer and absorption mechanisms are based on QM/MM approaches, which in turn heavily rely on TD-DFT. Yet, post Hartree–Fock methods start now to be considered in such studies, especially thanks to strong improvement in hardware efficiency. We recall here the two main problems related to the use of TD-DFT: Inability to properly describe charge transfer (CT) states and the impossibility of having multiconfigurational character. The CT problem can be strongly reduced by introducing long-range corrected functionals, while the latter requires the use of multi-reference methods, such as MRCI-DFT [88]. MRCI-DFT has been successfully applied to describe the peridinin carotenoid present in LH, for which even the slightest fluctuation in the bond length amplitude results in significant modification of the character of the excited states [89].

Despite the common use of accurate non-DFT QM methods for benchmark purposes [90], application to PPCs started to appear only in the last few years. A complete active space method combined with perturbation theory (CASPT2) has been recently used to study bacteriochlorophylls of LH2, to determine the geometrical variations affecting the absorption spectrum, and in particular the causes of the observed blue-shift [91]. The authors considered the highest-quality multistate multiconfiguration restricted active space with second-order perturbation theory correction (MS-RASPT2) method to compute excitation energies, combined with TD-DFT. With the combination of these two approaches, the authors were able to obtain high accuracy and speed in computation. They found that the changes in geometry of selected torsional angles are responsible for the blue-shift observed, due to the modification of the chlorophyll curvature. This is a consequence of the presence of different protein residues in proximity of the pigment, which push and pull it, regulating the excitation energy.

Another approach is to combine the approximate second-order coupled cluster (CC2) with reduced virtual space (RVS) [92,93] and the algebraic diagrammatic construction through second-order (ADC(2)) [94,95], which has been applied to study chlorophyll pigments of PSI [96]. In this study, the authors computed the lowest excited states of chlorophyll dimers, showing that the excitonic and electrostatic coupling are responsible for the red-shift observed in the absorption spectra. More interesting, the comparison with TD-DFT demonstrate how the later has difficulties to provide the correct shift in excitation energies, especially for excitonically coupled pigments.

A final approach consists in the use of the Bethe–Salpeter equation coupled with the GW approximation (BSE/GW), which has been successfully used to compute the EET of pigments found in the PE545 complex [97]. The suggested methodology has the advantage of being applicable with high precision for both molecular and periodic systems, opening to the possibility of studying systems with various sizes. Focusing on model dimers, the authors found that their approach reproduces almost exactly the experimental excitation energies, despite the approximations used (dimer pairs and no environment considered). The advantage of this method over TD-DFT is that BSE can accurately describe the CT excitations as the long-range Coulomb interaction is fully considered [98,99,100]. In addition, the electronic coupling calculations of the dimers lead to a small error compared to the supermolecule coupling, up to 5–10% for the absorption peak, suggesting that PBE/GW is a good method for the EET study.

As final remark, we would like to remind that the computational cost of the presented techniques should also be taken into account, as it is strongly dependent on the particular method used. As an example, we report here that the use of polarizable MM for QM/MM calculations can slow down the computation time up to 8–10 times compared to the use of electrostatic embedding. In addition, both numerical and post-HF methods request high computational efficiency which can be provided with high computing resources. Thus, a fine balance between accuracy of the method which is chosen and computational time needed to reach the desired accuracy is still requested and should not be overlooked.

### 2.4. Triplet–Triplet Energy Transfer 

Apart from ET present in natural photosynthetic apparatus, through singlet states, the triplet–triplet energy transfer (TTET) also plays an important role [101]. As discussed in the previous section, water plays a crucial role in mediating ET, yet only few investigations has been reported on its effect on TTET [102,103,104]. The same parameters governing the ET are also involved in the TTET process, based on the Dexter exchange mechanism [105] in which the rate constant, in the weak coupling limit is defined as [106]:

(6) $$k_{\mathrm{TTET}}=\frac{2\pi }{\hbar }{\left|V_{ij}\right|}^{2}\delta \left(E_{i}-E_{j}\right)$$

where δ is a function accounting for energy conservation during the transition and *V_ij_* is the electronic coupling between the triplet states of the donor and acceptor, which is defined as:

(7) Vij=HDA−1/2(ED+EA)SDA1−SDA2

where *E*_A_ and *E*_D_ are the diabatic energy of the acceptor and donor, *S* is the overlap and *H* the Hamiltonian between D and A. We would like to remind here that the spin is conserved between the initial and final state of the transition (^3^DA→D^3^A) and the forbidden nature of the spin makes the Coulombic term of the coupling null.

As TTET involved the simultaneous exchange of two electrons of different spin between the lowest unoccupied molecular orbitals (LUMO) and the highest occupied molecular orbitals (HOMO), its rate depends on the two overlap integral product, explaining why the exponential decay of TTET with the distance is twice that of ET [107]. Since this process is important at shorter distances with respect to ET, the presence of a bridge molecule (i.e., structural water, amino acid residue) can extend the overlap between the donor and acceptor wavefunction, thus enhancing the energy transfer, as described in the superexchange model [108,109]. This model allows the virtual population of triplet state localized on the bridge [110]. In this view, the donor–bridge, bridge–acceptor distance, their relative orientation and distribution, and the nodal pattern of the orbital involved in the ET affect the strength of the coupling.

This mechanism is of outmost importance, as it is crucial for the photoprotection from singlet oxygen formation. Interestingly, different LH systems present different TTET timescales, from nanosecond in purple bacteria LHC [111] to sub-nanosecond timescale for LHC of oxygenic organisms [112], and the mechanism underneath this difference is still not fully understood. It has been suggested that the ultrafast TTET occurring between the chlorophyll and carotenoid molecules leads to a localization of the triplet state over the carotenoid, affecting the electronic transition of the chlorophyll [113] and subsequent perturbations in the absorption spectra. It is thus of interest to determine the role of the electronic structure underlying the electronic coupling observed in the TTET. Computation can assess structural features underlying the TTET process, and in addition provide evidence of spin localization, which his of importance to enhance the stability of the system.

**Figure 9:**
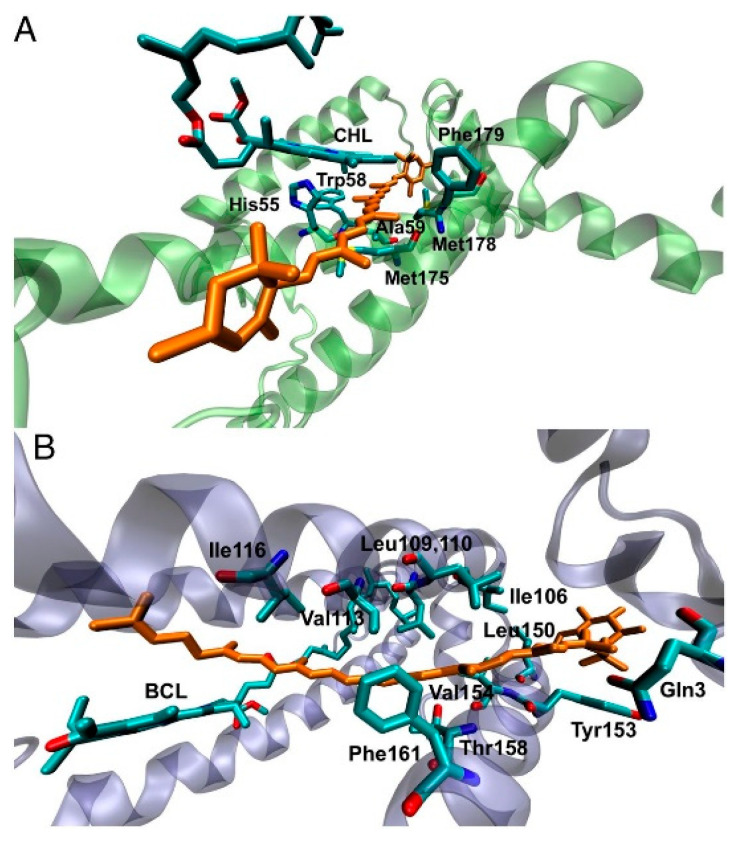
(**A**) Structural model of lutein 2 (orange) in LHCII monomer. (**B**) Structural model of rhodopin glucoside (orange) in LH2. The proximal (bacterio) chlorophyll, and amino acid residues that are within 3.5 Å of lutein are shown. These residues make up the QM layer in the QM/MM simulations. Reproduced from [114] with permission from National Academy of Sciences, 2017.

QM/MM method have been applied to study both the LHC-II and LH2 structures (Figure 9) by considering the ONIOM formalism, describing the Chl/carotenoid pair at the DFT level and the environment with the MM AMBER force field [114]. In this study the authors observed that the interchromophore interactions for closely interacting subunit pairs are responsible for the triplet state geometry perturbation, which is at the origin of the Raman shift experimentally observed [114]. In details, QM/MM calculations indicate that after the TTET, when the chromophore pair is in the Chl(S_0_)Car(T_1_) electronic state, the geometry of the carotenoid changes enough to give rise to the resonance Raman signal. An additional change in the carotenoid tilt relative to the tetrapyrrole when in its triplet state decreases the electronic coupling, ensuring that the triplet is localized only over the carotenoid without the involvement of any spin transfer. This effect, present in natural LHCs decreases the probability of side chemical reactions that can arise from the CT states, such as the formation of singlet oxygen species.

The TTET mechanism has been investigated also for the peridinin–chlorophyll a–protein (PCP) complex, as it reaches unity [115]. In this work, the authors employed a similar ONIOM scheme as previously discussed, but now the MM part is describe with the UFF force field, and explicitly consider one molecule of structural water between the Chl/peridinin pair. The orientation and position of this water molecule is imposed by its interactions with the surrounding chromophore pair as well as the closest amino acid residue. This computation shows that the water molecule is coordinated to the Mg metal of the chlorophyll. Triplet spin density calculations indicate that the low-lying triplet state is localized over the peridinin subunit, with a small but not negligible contribution over the oxygen atom of the water molecule. Once more, these results strongly indicate the direct involvement of water on the TTET mechanism, favoring the process by enhancing the overlap between the chromophore pairs.

## 3. Protein ET Mechanisms

Electron transfer (ET) is one of the most fundamental process taking place in biological systems, in particular when dealing with photosynthesis and respiration [116,117,118]. The redox center of proteins plays here an important role and its coupling with the surrounding protein environment affect the ET abilities. The rate of ET process is described by the semiclassical Marcus theory for nonadiabatic electron transfer [106]:

(8) $$k=\;\frac{2\pi }{\hbar }V^{2}\frac{1}{\sqrt{4\pi \lambda kT}}\exp\left(\frac{-{\left(\Delta G+\lambda \right)}^{2}}{4\lambda kT}\right)$$

where the key parameters controlling the ET rate are the electronic coupling V, the reorganization energy λ and the driving force ΔG. The electronic coupling exponentially decays with the distance *R* as:

(9) $$V\;\sim \;V^{0}\exp\left[-\frac{\beta }{2}\left(R-R_{0}\right)\right].$$

The difference in the oxidation and reduction midpoint potentials determine the thermodynamic driving force [119]; λ is the energy difference between reactant and product states and the gap between these electronic states at the intersection point can determine the ET mechanism. In fact, if the gap is large, the crossover probability is one, leading to a tunneling ET mechanism, while when the gap is small the ET can occur via hopping mechanism. The electronic coupling *V* describes the degree of overlap of the reactant and product wavefunctions. As the coupling follows Equation (9), the distance between the donor and acceptor has a strong impact on the ET process, as well as the nature of the environment (quantified by the decay factor *β*).

An alternative approach has been proposed by Moser and Dutton, adopting the Hopfield’s semiclassical rate expression [120,121]:

(10) $$\log k_{\mathrm{ET}}=13-\left(1.2-0.8\rho \right)\left(R-R_{0}\right)$$

which is a tunneling expression which leads to good accuracy when adjusted to an optimal driving force value ΔG= −λ. ρ is the packing density of the medium which equals the fraction of the volume in between the chromophores within the van der Waals radius of intervening atoms, and *R*_0_ is the vdW contact distance. From this expression is possible to obtain the decay factor *β* as:

(11) β=ln10(1.2−0.8ρ)

This packing density model is able to describe the physics of the tunneling pathway model, which complements the Marcus theory [122].

To understand the ET mechanism, knowledge of the electrochemical properties of the investigated systems are essential. Moreover, the nature of the protein is a determinant aspect in defining the ET process. As the protein is an heterogeneous matrix, local changes in proximity of the redox center might have strong impact on the ET ability of the system [123]. Commonly, two approaches are used to determine the effect of the protein on ET; (i) pathway (through bond tunneling), in which the decay constant *β* is determined for each through-bond and through-space segments and *V* is proportional to the product of all the *β* values; (ii) direct approach, in which *β* is related to the donor–acceptor distance and electrons “hops” through space [120,121]. Evolution perfected this process as proteins have tuned their environment to maximize the ET efficiency. Yet, a critical parameters remain the ET distance; there is a limit to the distance over which ET can occur at a rate sufficient to support biological activity. Nature favors proteins in which the average distance between redox centers is within 1.4 nm, for which the ET is robust via a tunneling mechanism, while for longer distances the ET flow is weaker and via a less effective hopping mechanism [121]. As the distance between active sites cannot be always rearranged, nature found the solution to shift the ET process from one-step tunneling to multi-steps hopping mechanism [124,125]. Tunneling through protein is mediated via amino acid residues, which merely act as conductive elements; on the other hand, during hopping some amino acids are reversibly reduced/oxidized and serve as intermediate states in between the donor and acceptor. As a result, if the intermediate state is reduced by the donor, consequently reducing the acceptor too, there is electron hopping. Conversely, if the intermediate state is oxidized by the acceptor leading to oxidization of the donor, there is hole hopping. Adding several intermediate states strongly decrease the donor–acceptor distance, replacing the long, slow tunneling into a cascade of short and fast ET hopping steps.

ET in Small Proteins

**Figure 10:**
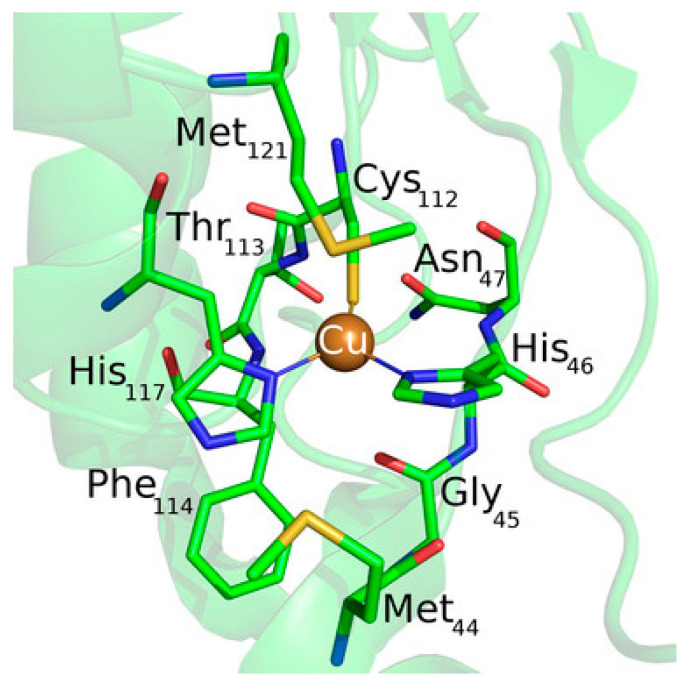
Active site structure of azurin as drawn from the 4AZU pdb file. Reprinted with permission from [140]. Copyright (2017) Wiley-VCH.

One of the most studied model system for ET mechanism is azurin (Figure 10). Despite not being a LHC, it is often used as model to compute the ET ability either in solvent or on surface, thus being a source of improvement for different computational methods. It is a small blue copper protein and serves as electron shuttles to mediate different ET reactions in biological metabolisms [119,126,127].

In order to assess the ET mechanism of any protein containing a redox center, its reduction potential should be computed with high accuracy, as is one of the key parameter needed for the ET. The computational determination of individual reduction potentials are affected by large systematic errors with respect to experiments, which make this task difficult to perform. Main components contributing to the reduction potential include electrostatics [128,129,130], metal–ligand interactions [131], hydrogen bonding interactions [132] and the constrain of the protein [133,134]. Common methods to compute the reduction potential consider MD simulations [135], continuum electrostatics methods [136], DFT [137,138], as well as hybrid QM/MM methods [139]. 

**Figure 11:**
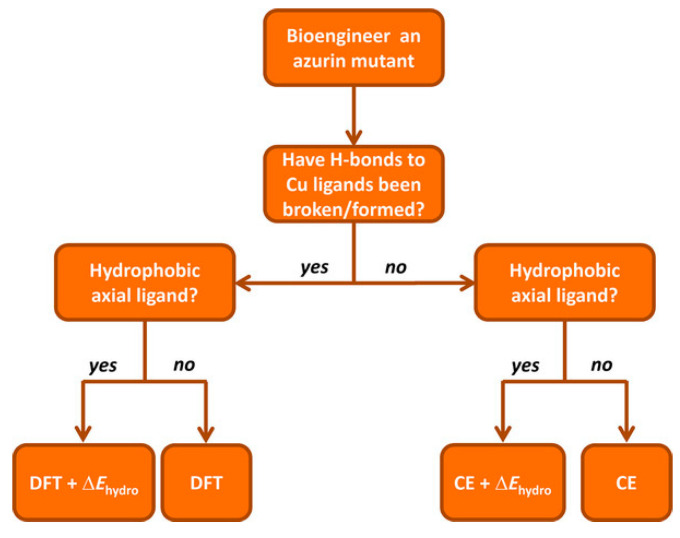
Flow diagram used to decide which computational method to use to compute the predicted reduction potential of an azurin mutant (CE = continuum electrostatics). Reprinted with permission from [140]. Copyright (2017) Wiley-VCH.

Recently, a comprehensive QM/MM approach for a large dataset of reduction potentials of mutants and an efficient and reliable computationally procedure has been reported [140]. The authors consider both continuum electrostatics and DFT methods on 24 azurin mutants, and found that the first gives good agreement with experimental observation despite its low computational cost and somehow high degree of approximations. Yet, it failed in properly describing the reduction potentials when explicitly hydrogen bonds are present and when hydrophobic axial ligands are considered. When hydrogen bonds are created/deleted through mutations, DFT or QM/MM approaches are needed. The introduction of hydrophobic ligand lead to a failure of DFT in predicting reduction potentials, and the authors suggest the introduction of a “hydrophobicity factor” through the Kyte–Doolittle hydrophobicity index ΔΚD [141]. In addition, when a combination of hydrogen bonds and hydrophobic axial ligands are present, DFT should be used to compute the reduction potential of the redox center (primary sphere) and the continuum electrostatic model for changes in the secondary sphere and the resulting total reduction potential is the sum of the two terms, in an approach similar to the ONIOM method. Finally, the authors reported an extensive prediction study on over 124 azurin mutants, for which they also provide a general scheme to be followed, in an elegant way, which might be used also for different PPCs (Figure 11).

As already discussed in the presented review, in order to accurately determine the redox properties of metalloproteins, both the electronic structure of the redox center and the environment must be considered. Treating the environment with an implicit solvent approximation eliminated the need of conformational sampling search, strongly reducing the computational cost [142,143]. Yet, these homogeneous models cannot be used to describe explicit contribution and coupling between the redox center and the environment, where polarization can play a crucial role [144]. Explicit residues surrounding the redox center and water molecules must be present, even in a minimal environment description. Once more, QM/MM is the method of choice also for ET studies.

In a recent study [145], QM/MM minimum free energy path (QM/MM-MFEP) method has been suggested to obtain accurate reduction potentials of azurin. In this approach the active site’s geometry is minimized on the potential mean force (PMF) surface of QM coordinates [146,147] and is additionally combined with the fractional number of electron (FNE) method for the study of redox reactions [148,149]. The mutual dependence of the QM and MM subsystems is iteratively solved in a self-consistent manner by performing the QM geometry optimization and the MM conformational sampling. After this first step, an interpolation of the reduced/oxidized QM states is performed and the FNE is used to efficiently connect the two redox states (the full procedure is reported in [145]). In this way the direct MD sampling is replaced by an iterative active site optimization, leading to a very good balance between accuracy and computational efficiency.

The authors obtained that the QM/MM-MFEP+FNE method is very accurate and efficient to describe the electronic structures of ET redox centers, when the anisotropic environment is explicitly considered and as input it needs only the protein structure. Moreover, the MD sampling performed is not dependent on the specific protein/solvent configuration, making this method general, transferable and able to handle systems with more than 1000 heavy atoms. In addition, the study shows that the presence of mutations (i.e., structural effect in axial ligand and outer sphere) are mostly electrostatic and additive, suggesting the possible use of this method to design novel redox centers for artificial photosynthesis. 

The central question in determining the ET ability of proteins from a computational point of view and also for the design of novel, highly efficient redox machines, is how much the environment affects the ET. If the protein provides a tunneling continuum shielding water from the active centers, the only variable to consider is the redox potential of cofactors (i.e., iron–sulfur clusters as present for PSI). As a consequence, the activation barrier is determined by the driving force and the reorganization energy for which a commonly assumed value range is λ ≃ 0.7–0.8 eV [121]. In addition, detailed studies have shown that the exponential decay *β* value depends on the secondary structure of the protein, and can vary in the *β* ≃1–1.4 Å^−1^ range [150,151]. Only the free energy or reaction (i.e., the driving force) is thus needed to calculate the ET rate. The small energy range of possible values for both *λ* and *β* put strong restrictions in the design of ET chains, resulting in efficient ET transfer for edge-to-edge distances not exceeding 14 Å [121,152]. In addition, studies on electron tunneling of different PPCs revealed that the presence of structural water occupying the protein pocket along the ET pathway strongly enhances the protein’s ET ability [153]. These results dismiss the previously exposed idea that the environment act as a generic medium and does not affect the local structure, as water can enhance the electronic communication between redox centers. This finding poses a new view on the role of the protein, which is not merely a passive scaffolding, and should be explicitly considered for computations. In fact, if structural water is present in the vicinity of redox centers, the reorganization energy might increase up to 1.2–5 eV depending on the amount of water present [154]. As a consequence, the classical view of the Marcus theory should be revisited, in order to account for these novel parameters.

Martin et al. [155,156] applied this novel concept on the analysis of ET in cytochrome *c*, another widely used small light harvesting protein in computation, explicitly accounting for structural water and employing the empirical valence–bond method as well as a simplified description for the linear anisotropic polarizability of the active site. The differences in redox states polarizabilities is a general mechanism applicable to all ET reactions, which become important when the polarizability change between redox sites become predominant. In this work the authors developed a formalism to compute the polarizability and electrostatic terms for the active state for an anisotropic system, considering the difference in environment surrounding the active site. The inclusion of this term results into coupling of the two terms due to the presence of the protein–water interface, which results into a significant decrease in activation barrier for ET. From these results, the authors concluded that the natural design of the active site of cytochrome *c* (cyt) is optimized for the presence of large fields which originate from the contact between the heme group and the water molecules. This results point out the importance of considering structural water molecules when performing computations, as it might strongly affect the description of ET process. It has been shown that the presence of water in proximity to the redox group has a significant impact in both ET kinetic [156,157] and protein functions [158,159,160]; this lead to the creation of strong interfacial heterogeneity which generates strong interfacial electric fields which, in turn, is responsible for the decrease in ET reaction barrier. These simulations show that the presence of both polar and charged groups on the cyt allow for the penetration of water molecules into confined regions of the protein itself, producing a highly polar environment which strongly affect the ET. Moreover, polarization must be included in computation to properly describe this effect. These studies provide a novel picture of the ET in protein, which is more specific than commonly assumed.

## 4. Protein/Surface Interactions

The realization of artificial photosynthesis devices often takes place in an all-solid state device in which the protein is directly or indirectly interacting with a metal surface, to control the ET flow [5,8,9]. In addition, often the LHC is interacting with another small protein which act as shuttle of electrons (such as cytochrome), facilitating the ET process. Thus, protein adsorption on a surface is an interesting challenge for computation, as many different interactions are present, such as hydration, conformational changes and orientation of the protein to enhance the ET to/from the surface. These aspects become important when the design of novel bio-compatible applications is considered, like for biosensors [161], drug delivery systems [162] and bio-hybrid materials [163]. Experimental observations of protein absorption on a substrate have focused mainly on morphology [164], structural deformation of the protein upon adsorption [165], the effect of the surface chemistry [166] as well as kinetic of ET [167]. Yet, it is still difficult to assess the single-protein microscopic details from experimental observation, as they mainly consider thin layers or aggregate states. Computation plays once more an important role, considering MD simulations, capable to provide not only atomistic details on the adsorbed configurations, but also the time evolution of few hundreds of nanoseconds, even up to microseconds for small proteins [168]. The adsorption process commonly involves the presence of a solvent (normally water) and the vast amount of molecules makes systematic studies still challenging. A common approach [169] consists of considering first an implicit solvent to determine the most energetically favorable protein orientations on the surface, followed by relaxation of the whole system including the explicit solvent molecules. Despite the reliability of this approach, it is still difficult to describe the whole absorption process from computation [170] and often this first step is skipped, with more focus oriented toward the description of the properties of the already formed protein–substrate interface, such as the intensity and direction of ET.

When immobilized on a surface, the ET occurs between the surface and redox center of the protein, giving rise to a transition from electronic to ionic charge transfer, as the electrons have to leave/enter the protein, causing the oxidation/reduction of the redox center, which is commonly observed by cyclic voltammetry measurements. This change in ionization of the protein is accompanied by changes in the environments (as it can be also charged, due to the ET process) and reduces the electrostatic barrier for subsequent steps by electrical screening [171]. If this process take places in a system in which the electrolyte is absent, or is not directly involved in the ET process, it is often termed as electron transport, and is generally considered in a solid state configuration [172]. As in this last process ions from the electrolyte are not present, charge balance is not possible and the whole process requires the electronic conduction across the whole protein to reach the electrode (or vice versa) which is defined as the electron flow through/across the protein which is in contact with the surface [173]. Computational approach to biomolecules adsorbed on surfaces can be strongly beneficial to experimentalists not only to unravel the ET mechanisms, but also to predict the behavior of biological systems with details that are not yet accessible by experimental techniques. To assess these processes at the interfaces, as for processes described in previous sections, a variety of computational methods and approaches can be considered, ranging from MD to sample the conformations of the protein adsorbed on the surface, to QM, QM/MM and ab-initio molecular dynamic (AIMD) to assess the ET and transport in both ground and excited states. For a comprehensive review on the strength and kind of different applicable methods, we refer to [122,174]. We would like to briefly remind in here how the photosynthetic devices are obtained by experiments. While the assembly is done in the presence of a solvated environment (i.e., water molecules with physiological concentration of ions), the final device is in a full-solid state, in which the solvent has been dried up. This brings additional challenges to computations, since now two different pathways can be considered to compute the adsorption and ET processes. In a first approach, in which the focus is the adsorption process, the protein and surface are immersed in a solvent environment, which is essential for the correct modeling of the process. In a second approach, in which the focus is on the DET, the protein is already considered adsorbed on the surface, and the solvent is neglected (apart from structural water molecules) to model the final experimental device.

### 4.1. Protein–(Self-Assembly Monolayer (SAM))–Metal Interface

MD is the method of choice when conformational studies are considered. Generally, these models consist of an idealized structure of the solid surface, which might not be representative of the actual experimental system, and simplifications on both the bond-topological structure and protein structure, protonation and interfacial solvent structure often affect the setting up of the MD simulations. Noble metals such as silver and gold are the most common metals used in experimental applications, like biosensors and bioelectronic electrodes [175,176]. The substrate is commonly considered crystalline, while in experiments often amorphous or polycrystalline surfaces are present. This latter case is especially interesting, since changing the adsorption facet of i.e., Au for a protein, can lead to strongly facet-dependent results [177]. In fact, for flat, planar Au surface, the facet of choice is the Au (111), as it is assumed to be the dominant one. While this is true for planar interfaces, more care must be payed when nanoparticles are considered, since there is still debates on which facet is the predominant one. Moreover, the presence of structural defects on the surface, as well as the presence of various functionalizations might have a strong impact on the protein conformations, and shall not be neglected when such analyses are planned from a computational side. 

In addition, often self-assembly monolayer (SAM) are present in between the metal and the protein. SAM are thin films of molecules which spontaneously adsorbed in an ordered assembly over the metal (often Au). Typically, SAM are chemisorbed on the substrate through their reactive anchoring group (i.e., thiol or silate) to form a very stable assembly, and present a tail group which can be functionalized by small organic groups or even proteins. Together with the length of the SAM forming molecule itself, these characteristics determine a change in the physico-chemical properties of the metal surface and can be adjusted accordingly to the desired application. Moreover, the presence of a SAM allows for specific absorption interaction with the protein, limiting and sometime imposing a specific orientation of the biological component on the SAM, which, in turn determine the ET ability of the interface. Finally, SAMs act as electrical insulators, passivating the metal surface [178], protecting if from degradation (i.e., in case of metal oxides).

**Figure 12:**
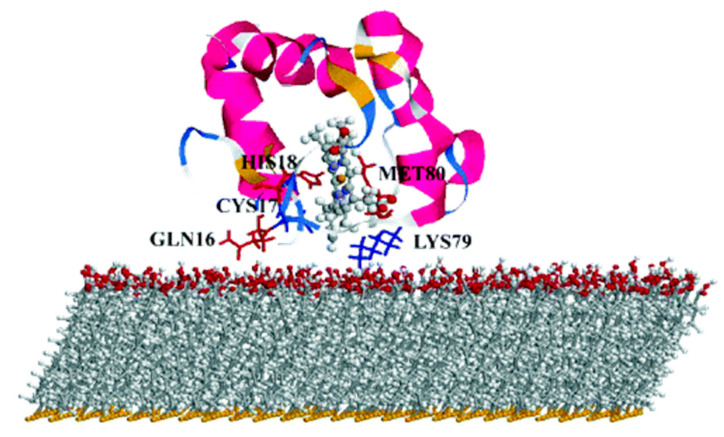
Simulation-predicted electron-transfer pathway of cyt on negatively charged surfaces. Reprinted with permission from [179]. Copyright (2004) American Chemical Society.

One well known system often use in computation is cytochrome *c* (cyt). Cyt plays an important role in a variety of artificial bio-applications like biosensors, bioelectronic devices and biofuel cells, and its adsorption on a SAM covered gold surface has been extensively studied. For these applications, the knowledge and ability of orienting the cyt on the SAM is crucial, as on it depends the ET ability of the whole interface. It has been reported that to obtain a fast ET, the protein should be oriented with the heme ring close to the SAM, in a perpendicular orientation [179] (Figure 12).

By mean of combined Monte Carlo and MD simulations, the authors of this work assessed the conformation and orientation of cyt adsorbed on a negatively charged carboxyl-terminated SAM-Au surface. Cyt could orient itself with the heme group perpendicular to the surface, forcing a specific direction of the dipole moment which is a key parameter on the determination of the final orientation. On the basis of these results, the authors indicate a possible ET pathway, from the iron of heme to the surface through a series of specific amino acids. Moreover, they warn the readers on the effect of a charged surface, which can be detrimental for the function of the protein itself.

**Figure 13:**
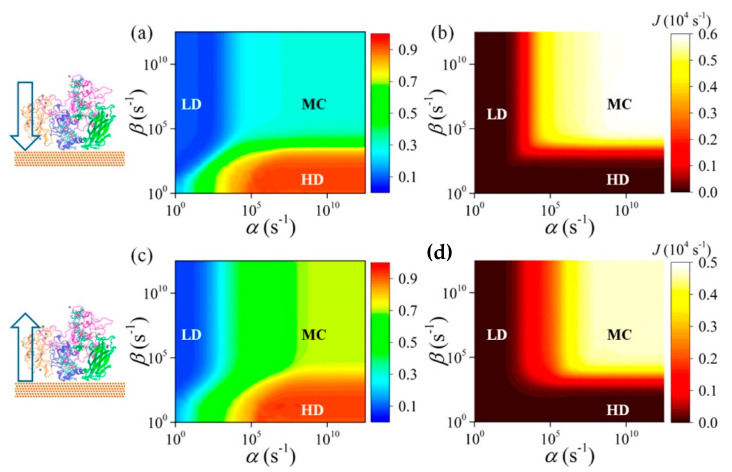
(**a**,**c**) Phase diagram of the time-averaged electron occupation density ⟨n⟩ for all 10 hemes in adsorbed structure as a function of the incoming (*α*) and outgoing (*β*) electron transfer (ET) rates for the transfer direction heme10-to-heme5 (from the environment to the surface through the protein) and heme5-to-heme10 (from the surface to the environment through the protein), respectively. (**b**,**d**) The corresponding phase diagrams of the net electron flux J of adsorbed protein for the transfer direction heme10-to-heme5 and heme5-to-heme10, respectively. Reprinted with permission from [185]. Copyright (2016) American Chemical Society.

Beside cytochrome, multiheme cytochrome (Mcyt) are a family of proteins in which two or more heme center are present, with Fe-Fe distance shorter than 1.55 nm [180,181]. Mcyt have the unique capability of long-range ET, even up to several micrometers [182], which can be applied for redox catalytic activities and electron storage [180,183]. Conversely to one heme group cyt which are widely study, the absorption on a surface and its relative ET of Mcyt is still not well understood. Mcyt can form a network of ET pathways which facilitate the total ET, and can have potential applications in i.e., solar-conversion and bioenergy [182,184]. Wei et al. [185] studied a decaheme cyt folded in four domains adsorbed on a gold substrate via thiol bonding interactions (Figure 13). The aim of their work was to assess which orientation and conformation of the adsorbed protein on gold will favor an efficient ET. Due to the complexity of the system studied, the authors devised a multiscale computational protocol in which full atomistic MD coupled with the molecular mechanics/Poison Boltzmann surface area (MM-PBSA) method are used to predict the configurations of the adsorbed protein on Au (111) surface. MM-PBSA has been shown to yield accurate prediction of the binding free energy of protein-surface interactions [186] and provides quantitative information on the driving and barrier forces of protein adsorption and docking. In the next step of their protocol, the authors perform Kinetic Monte Carlo (KMC) simulations [187] to study the ET across the protein and the hopping of electrons among the Fe atoms of the heme groups and their transfer to the surface. ET rate was quantified by using the nonadiabatic rate equation from the Marcus theory and the total ET flow assessed (Figure 13) [188].

As a result of this protocol, the authors were able to predict the Mcyt adsorption and ET rate when interacting with a flat gold surface. They demonstrated that the orientation of the protein, which in turn controls the ET flow, is controlled by the dehydration of the surface. Moreover, they found that this flow is more effective when the electrons move towards the interface, while is smaller in the reverse direction.

Another widely used PPC in computational studied is azurin, yet only few studies reported a computational protocol when adsorbed on a metallic surface [171,189]. Once again, MD is the method of choice to assess the conformation and orientation of the protein on the surface, as well as the retention of its folding upon adsorption. Ortega et al. [190] performed the study on an azurin (and several single amino acid mutations) adsorbed on gold surface without specific interaction, by mean of long MD simulations (up to 0.5 μs) to describe the structural changes and dynamic of azurin adsorption. The authors found that the presence of a single amino acid mutation quenches the flexibility of some region of the protein, making it stiffer. This increase in stiffness affects the adsorption dynamics on gold; the wild type adsorbs with two preferential configurations (lying-down and anchored via hydrophobic parch) thanks to the higher mobility which allows for a reorientation of the structure during the adsorption process. On the other hand, the stiffer mutants cannot easily reorient, and the final adsorption geometry is strongly dependent on the initial orientations, affecting the stability of the interface and, in turn, the ET efficiency.

### 4.2. Adsorption on Low Dimensional Materials

Low 1D and 2D dimensional materials are emerging as promising candidates not only as support layers due to their peculiar electronic properties, but also for active center for charge separation and storage [191]. The archetype for this materials are carbon allotropes, in particular carbon nanotubes (CNT) and single layer graphene (SLG) and due to their extremely high surface area, they are optimal candidates for the design of highly efficient biosensor applications [192]. The unique electronic characteristics of these materials is the presence of a strongly delocalized π electrons clouds which strongly favor the creation of van der Waals interactions with organic molecules and proteins.

As for the previous section, computational approaches and methods has been mainly used to describe protein/low dimensional material interaction in terms of MD simulations, to describe the adsorption process. In the last few years, rigorous approaches have been developed in order to meet the strong demands of conformational samplings [193] needed for this kind of protein/material interactions since they are often intrinsically disordered. Moreover, the size of the system pushes classical MD to its boundary, and to have meaningful results, MD should be long enough to let the protein fully adsorb and relax on the surface. To overcome these bottlenecks, Walsh et al. developed an “economical” polarizable all-atom force field to describe a graphene/water/peptide interface used in combination with replica exchange solute tempering MD (REST-MD) simulations [194]. This method is based on a replica exchange Hamiltonian-based approach that allows for efficient conformational sampling of complex interfaces. As results, the authors found that a strong binding is obtained not only for aromatic residues of the peptide, but also for residues possessing amide groups, like Asn and Gln, of a short peptide sequence. This study was performed in diluted solution for one (short) peptide chain, in order to avoid peptide-peptide interactions, which might complicate the description of the force field. In a subsequent study [195], the same authors considered the presence of multiple peptides to study their aggregation and organization on SLG/water interface, for the first time in an all-atom approach focused on advanced conformational sampling. They concluded that despite the presence of a considerable degree of peptide-peptide interactions, the interaction with the surface still resemble the one obtained from the monopeptide system, without leading to self-organization.

Moving a step forward toward complexity, Kim et al. investigated the adsorption of a peptide on SLG supported on gold as well as on multilayer graphene [196]. The result of this study was the negligible influence of the supporting layers (either gold or SLG) on the adsorption properties of short peptides. Yet, the authors did not consider the SLG/Au interactions explicitly, which might be important in the determination of the adsorption process. Challenges are still present when modeling these interfaces, such as how to consider the commensuration of the SLG/Au lattice parameters and to what point a mismatch between the two lattices is acceptable. 

As already shown in previous Section 3 and Section 4.1, MD simulations strongly focus on the adsorption of proteins on different substrates. These simulations address the problem in two ways; (1) the study of the dynamic of the adsorption process of a (small) protein or peptide sequence at liquid/solid interfaces [197,198], in which the process is divided into three phases, namely the (biased) diffusion, the anchoring to the surface and the lockdown (or relaxation) phase. (2) The study of already adsorbed protein on a surface using thermodynamic methods, in which different interactions such as hydrophobic, π−π staking or hydrogen bond play an important role. The process behind the adsorption mechanism is nowadays fully described and supported by robust demonstrations [199], yet it is based on short peptide sequences and not on proteins. On the other hand, the protein/surface interaction is specific for each protein/substrate pair, and depends on both the nature of the protein and the specific surface composition. Moreover, the relationship between the structure of the adsorbed protein and its function (i.e., is the function retained after adsorption? Is there a relation between the adsorbed state and the ET?) is still not clear.

In a recent study, Zhang et al. focused on the adsorption mechanism of cyt c on CNT by mean of MD simulations [200], and in particular on the dynamic of the process when water is present. The authors considered a double layer of water molecules in between CNT and the protein and characterize the dynamic of adsorption of cyt considering 64 initial orientations over CNT. They found that water molecules strongly affect long-range electrostatic interactions which in turn, affects the adsorption of cyt. In this process, once the protein is anchored to the surface, a lockdown phase in which water is repelled from CNT takes place, in order to obtain stable conformations. Once more, this study shows that water molecules should not be neglected in simulations, either when ET or adsorption dynamic process are considered. Moreover, they described the relationship between the adsorption conformation and the ET by considering the distance and tilting of the heme group from the CNT surface. They consider the ET through bond mechanism, in which electrons flow form the iron of heme to CNT is maximized when the heme is perpendicular to the surface [201] and is strongly dependent on the relative distance between Fe and CNT.

Graphene and its soluble derivative graphene oxide (GO) hold great promises also for the creation of biological interfaces and can be considered as materials of choice for the regulation of ET of adsorbed proteins [202,203,204], not only due to their peculiar electronic properties, but also to the high surface area. It has been reported that these materials can accommodate a vast range of redox proteins and facilitate a rapid ET through the layered materials [205]. Moreover, adsorption of cyt c over GO/reduced GO (rGO) resulted in a modulation of the protein activity due to the changes in the heme micro environment caused by the different interactions between the protein and the materials [206]. Zhao et al. [207] reported the study of cyt c adsorption over graphene and GO by mean of parallel tempered Monte Carlo (PTMC) algorithm coupled with MD simulations to assess the orientation of protein on the surface, the nature of the interactions, the subsequent conformational changes and the ET pathway. With the PTCM method, cyt c is considered in a united-residue model in which each amino acid is reduced to a site centered interaction at the carbon alpha, and the structure of the whole protein is kept rigid. The graphene/GO surface is regarded structureless and the interactions with the protein described with both vdW and electrostatic interactions. At the end of this process, optimal orientations are obtained and the most favorable were chosen as input structures for MD simulations. This protocol replaces the traditional docking procedure which is commonly performed when protein–protein interactions are present, but fails for heterogeneous interfaces as the one reported here.

**Figure 14:**
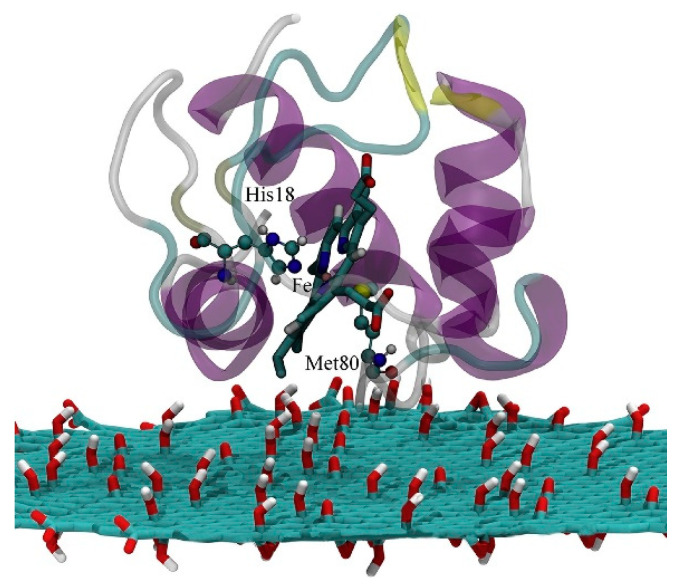
The electron transfer pathway of Cyt c on the graphene oxide (GO) surface. Reprinted with permission from [207]. Copyright (2018) Elsevier.

From MD simulations the authors obtained that hydrophobic interactions determine the cyt-surface adsorption, with the heme group parallel to the graphene surface and almost perpendicular when adsorbed over GO (which in this study was negatively charged). The authors concluded that ET for the cyt/graphene interface is thus inhibited, while it is enhanced for the cyt/GO interface. Following recent studies showing that the ET of immobilized cyt is controlled by the interplay between tunneling probability and protein dynamics [201], the authors defined key parameters for the ET process: Redox center location, orientation of the protein on the surface and ET distance. From this study, the authors obtained that in order to favor ET tunneling (which exponentially decays with the distance) the minimal distance between the heme iron atom and the surface should be not higher than 1.3 nm. They obtain a similar distance for the protein adsorbed on GO, which should show efficient ET (Figure 14), while should be less pronounced when adsorbed on graphene, as the distance is now increased up to 1.8 nm. They rationalize these results by considering the different orientation of the heme group relative to the surface.

Another study on the adsorption of cyt c_553_ on graphene has been performed combining MD and QM approaches to assess both the orientation and the ET ability of the surface [208]. The authors performed docking to obtain the most favorable orientations of the protein over SLG, and considered the three most stable ones as input for MD simulations. The results show that the three structures are thermodynamically stable, and the heme is oriented with different tilt angles and distances with respect to SLG for all of them, which should strongly affect the ET ability of the interfaces. In this work, the authors considered a different approach to assess the ET flow, namely to compute the transfer integral (which is proportional to the electronic coupling) and obtained net ET flow from SLG to the heme group independently form the orientation or distance of the two fragments, but with magnitude strongly dependent on the distance, as previously reported for the tunneling ET mechanism, as well as the tilting, showing highest ET efficiency when heme is (almost) parallel to the SLG surface. 

Apart from carbon based low dimensional materials, other structures of interest to form bio-interfaces are hexagonal boron-nitride (h-BN) and MoS_2_. The first material can be considered as insulator, while the second is a semimetal. These materials are relatively novel, and little is present in literature on their possible protein adsorption abilities and relative ET mechanism. Thus, a substantial increase in the knowledge of biomolecular adsorption at these interfaces is required to control the formation of these hybrid systems.

Several works on a simple water/h-BN interface in which novel force field are developed and validated have been reported in literature [209,210], but only a few MD simulations are present up to date for ternary protein/water/h-BN structures. The main reason behind this is the lack of experimental data to validate the force fields for biomolecular adsorbates. One of these study reports the adsorption of short peptides on h-BN nanotubes and nanosheets [211]. The authors performed MD simulations using a combination of Lennard-Jones parameters for the water/surface interface, and reported the structural deformations of these peptides once adsorbed. Due to the lack of experimental data, this force field combination still needs to be validated, but it is a first attempt to push the field towards novel horizons. 

Literature on MoS_2_ based hybrid bio-interfaces are even scarcer, both experimentally and computationally. A recent report present MD simulations in which combining rules are used to generate the interfacial terms for the force field for bio-MoS_2_ interfaces, leading to some inconsistency compared to experimental findings [212]. The lack of tuned force field for these interfaces is the main cause for this mismatch, and fundamental studies are thus needed for these interfaces, especially to verify the force field terms. Yet, despite the need of such data, even QM reports are scarce. Structure binding energies of single amino acids on MoS_2_ have been reported using periodic DFT [213]. Interfacial force field terms based on first principle calculations start to appear in literature, especially for peptide/MoS_2_ interfaces [214,215], but they still rely on mixing rules, and their verification is needed to ensure that the outcome of MD simulations is robust and correctly interpreted. As an example, Gu et al. [216] reported refinements in the Lennard–Jones parameters for the water/surface interactions, as the use of mixing rules resulted into too strong interactions when applied for the adsorption of a peptide. Yet, the need of obtaining reliable force field parameters beyond the mixing rule is still strong, and can be one direction to follow for the advancement of computation for hybrid interfaces to consider different low dimensional materials beyond graphene and its derivatives. 

## 5. Conclusions and Perspectives

In this review we have shown that while the computational methods for the study of absorption properties of complex LH systems are mature, with many different multiscale approaches been developed over the year, the ET process are still poorly studied, for both protein in a solvent and when adsorbed on a surface. For sure the size of the systems is one of the main bottleneck for the computation of ET processes, but important developments are on the way. 

The main focus of computation in this respect is now on the adsorption process, considering a multiscale approach based on MD and KMC to assess the ET ability of the complex systems, but without any ab initio quantification. Moreover, the size of the whole interface is often close to the computational capability of MD simulations, resulting inefficient due to short sampling time and different approaches such as CG should be considered. A shift towards more robust methods (involving fewer approximations) is thus needed, and maybe in the next few years we will see a change in this trend, in which novel multiscale approaches will be developed to quantitatively describe this challenging process. In particular, the widely use QM/MM approach which is the backbone for light absorption simulations, can become the method of choice also for the ET studies, since allows for the computational analyses of different optical and transport properties without neglecting the environment, which is an essential part which should always be present to obtain reliable results. Yet, within this approach some caution must be considered, as the widely used TDDFT method is not able to reproduce charge transfer states and might lead to questionable results, but steps towards different methods have been developed and are showing their potential. This is true especially when considering proteins into a biological environment, but still far to become a common practice when proteins are adsorbed on a surface to create a hybrid bio-interface. 

Protein adsorption on surface is still mainly accessed by classical MD simulation in order to describe the dynamic of the process itself and the conformational changes of the protein upon adsorption. Moreover, the use of accelerated GPU cards might bridge the gap between the size of the system and the ability of sampling the conformational space in a (almost) complete manner. The ET process is still out or reach, but it can be explored in the next few years with the application of a multiscale approach which has been successfully used for the description of optical properties of these biological system. Considerable advances have been already made, but many challenges are still present. In particular, further effort is needed to model the protein adsorption on realistic surface coverages and with the presence of surface defects, like heterogeneity, which can strongly affect not only the conformation of the protein (or SAM) on the surface, but in turn the ET ability of the whole interface. It is in here that the multiscale approach can show its potential, allowing to study complex interfaces not only from a conformational point of view, but also optical and transport properties of model interfaces which are closer to the experimental counterpart, without the need of drastic approximations and simplifications.

Recently, the computation of protein adsorbed on low dimensional materials started to be of interest, but is still not fully exploited despite the readiness of computational approaches to describe these interfaces, in particular when graphene and its derivative are considered. The presence of a supporting substrate is still a challenge for MD simulations, especially due to the lattice mismatch often observed, as well as the presence of proper interfacial force field parameters to describe these heterogeneous interfaces. For low-dimensional materials the main focus till now is localized over the adsorption process, while the deep understanding of the ET process is still qualitative, and more effort should be made along this direction, taking advantage of a multiscale approach.

Considering 2D materials beyond graphene and its derivatives, the field is in its infancy, and many opportunity can be foreseen. The first, pressing challenge is to obtain in a rigorous way novel force field for these heterogeneous interfaces, which would lead to strong advances in this area. New classes of low dimensional materials are relatively under investigated for hybrid bio-interfaces, and provide an attractive direction for expanding and improving computation.
